# IL-10 Blocks the Development of Resistance to Re-Infection with *Schistosoma mansoni*

**DOI:** 10.1371/journal.ppat.1002171

**Published:** 2011-08-04

**Authors:** Mark S. Wilson, Allen W. Cheever, Sandra D. White, Robert W. Thompson, Thomas A. Wynn

**Affiliations:** 1 Immunopathogensis Section, Laboratory of Parasitic Diseases, National Institutes of Allergy and Infectious Disease, National Institutes of Health, Bethesda, Marlyand, United States of America; 2 Biomedical Research Institute, Rockville, Maryland, United States of America; Trudeau Institute, United States of America

## Abstract

Despite effective chemotherapy to treat schistosome infections, re-infection rates are extremely high. Resistance to reinfection can develop, however it typically takes several years following numerous rounds of treatment and re-infection, and often develops in only a small cohort of individuals. Using a well-established and highly permissive mouse model, we investigated whether immunoregulatory mechanisms influence the development of resistance. Following Praziquantel (PZQ) treatment of *S. mansoni* infected mice we observed a significant and mixed anti-worm response, characterized by Th1, Th2 and Th17 responses. Despite the elevated anti-worm response in PBMC's, liver, spleen and mesenteric lymph nodes, this did not confer any protection from a secondary challenge infection. Because a significant increase in IL-10-producing CD4^+^CD44^+^CD25^+^GITR^+^ lymphocytes was observed, we hypothesised that IL-10 was obstructing the development of resistance. Blockade of IL-10 combined with PZQ treatment afforded a greater than 50% reduction in parasite establishment during reinfection, compared to PZQ treatment alone, indicating that IL-10 obstructs the development of acquired resistance. Markedly enhanced Th1, Th2 and Th17 responses, worm-specific IgG1, IgG2b and IgE and circulating eosinophils characterized the protection. This study demonstrates that blocking IL-10 signalling during PZQ treatment can facilitate the development of protective immunity and provide a highly effective strategy to protect against reinfection with *S. mansoni*.

## Author Summary

Schistosomes are zoonotic parasitic helminths that infect hundreds of millions of people worldwide. Despite effective chemotherapy, schistosomiasis- the disease caused by these parasites, still plagues tropical regions of the world. This is due, in part, to poor resistance to reinfection resulting in high re-infection rates following treatment. This lack of resistance is intriguing, as effective treatment relies upon drug-induced parasite damage combined with host immune mediated killing. Furthermore, it has been widely reported that post-treatment, individuals develop and retain elevated levels of anti-parasite immune responses. We therefore asked why resistance to re-infection is so poor, despite the development of significant anti-worm responses post-treatment.

It is essential that immune responses are controlled by various immunosuppressive mechanisms to prevent immune-mediated pathologies. However, a robust immunoregulatory response may obstruct the development of protective immunity. Thus, a balanced immune response providing a non-pathogenic yet effective immune response may be required for the development of effective resistance to reinfection. Understanding the immunological mechanisms of resistance to re-infection and the role of effector and regulatory responses may aid in the development of more effective vaccines and treatment strategies for schistosomaisis. This study suggests that combining chemotherapy with drugs that block IL-10 might provide an improved strategy to elicit acquired immunity to this parasite.

## Introduction

Despite inexpensive and effective chemotherapy, schistosomiasis continues to plague sub-Saharan Africa, south-east Asia, south America and areas of the Caribbean. This is due, in part, to rapid re-infection rates following curative chemotherapy. Recent estimates indicate that 200 million individuals are infected with a further 700 million people at risk of infection [1]. Although schistosomiasis research and control programs [2], [3], [4] are attempting to increase the availability and variety of anti-schistosomal drugs [5], [6] (particularly following the emergence of praziquantel (PZQ)-resistant parasites [7], [8]) and develop novel vaccines [9], [10], [11], [12], additional approaches are required to complement classical chemotherapy.

A central tenet of vaccine strategies for schistosomiasis is the induction of immunity to infection and re-infection. Despite significant work in this area over the last half century, the precise mechanisms of resistance to re-infection continue to be debated. Results from sufficiently powered longitudinal field studies suggest that resistance to re-infection can develop naturally [13], but only after multiple rounds of exposure, treatment, and re-infection [14], [15]. These observations have led to an age-dependent resistance model [14], [16], which has dominated the schistosomiasis field for several decades. In this model, children <15 years old are highly susceptible to re-infection post-treatment, with a gradual increase in resistance over time [16], [17]. However, a recent study by Karanja and colleagues[18] challenged this model and reported that occupationally exposed individuals in Kenya fall into one of three distinct groups; (1) immunologically resistant to re-infection independent of age; (2) immunologically susceptible irrespective of age; and (3) resistant to re-infection following multiple rounds of treatment. This important study suggested that resistance to re-infection could develop in response to curative chemotherapy and thus may not be strictly age-dependent, but rather exposure-dependent. It is worth noting however, that age and exposure are themselves proportional to one another and closely related.

Many studies have reported correlations between immunological responsiveness to schistosome antigens and protection from re-infection, supporting an immunological mechanism of resistance to re-infection. Karanja and colleagues [18] support this hypothesis and implicated CD4^+^T cells in the development of resistance, loosely inferred from reduced resistance to schistosome infection observed in HIV^+^ patients with low CD4 counts. To date, few reports have provided a detailed analysis of concurrent immunological effector and regulatory responses that are induced following PZQ treatment, either in humans or animal models. We wondered whether activation of inappropriate ‘regulatory’ responses might be disrupting the development of resistance to re-infection following curative chemotherapy. To address this question, we conducted a detailed analysis of the immunological response post-PZQ treatment, characterizing both effector and immunoregulatory responses in the highly permissive experimental mouse model of *Schistosoma mansoni* infection. In addition to enhanced Th2 responses as previously reported[19], [20], we observed increased in anti-worm Th1 and Th17 responses two-weeks after PZQ treatment. Despite the exaggerated anti-worm responses in PZQ-treated mice, no resistance to a subsequent challenge infection was observed. Using a bicistronic IL-10^gfp^-reporter mouse system, we identified increased populations of CD4^+^CD44^+^CD25^+^GITR^+^IL-10^gfp+^ cells in the blood, mesenteric lymph nodes, spleen and liver of PZQ-treated and re-challenged mice. These observations suggested that an effector or regulatory population expressing IL-10 might be restricting the emergence of immunity following PZQ treatment. To investigate this, we used anti-IL-10R antibodies to block IL-10 signaling. Mice administered anti-IL-10R antibodies during PZQ-treatment displayed a greater than 50% reduction in worm burdens compared to control mice. Taken together, these data indicate that IL-10 signaling impedes the development of immunity to *S. mansoni* and suggests that interfering with immunoregulatory mechanisms in combination with PZQ can accelerate resistance to re-infection in mice.

## Methods

### Animals

Six to eight week old female C57BL/6 and C57BL/6 Foxp3^gfp^ reporter mice, originally provided by Bettelli and colleagues [21], were maintained by Taconic farms. C57BL/6 IL-10^gfp^ ‘tiger’ mice were kindly provided by Dr. Richard Flavell [22]. All animals were housed under specific pathogen-free conditions at the National Institutes of Health in an American Association for the Accreditation of Laboratory Animal Care–approved facility. A minimum of 7 mice were used in each experimental group unless otherwise indicated.

### Infections and reagents

Mice were infected percutaneously via the tail with 35 or 120 cercariae, as indicated, with a Puerto Rican strain of *S. mansoni* (NMRI) obtained from *Biomphalria glabrata* snails (Biomedical Research Institute). Where indicated, cercariae were attenuated with 40 krad of gamma irradiation from a ^137^Cs source. Mice were vaccinated by immersion of their tails in water containing approximately 500 attenuated parasites for 40 minutes. SEA was obtained from purified and homogenized *S. mansoni* eggs as previously described [23]. Animals were perfused at sacrifice so that worm burdens could be determined. Two 500 mg/kg doses of Praziquantel (PZQ) (Sigma Aldrich, St. Louis, MO) were administered in a Glycerol/Cremaphor EL emulsion to infected mice at indicated times by oral gavage. One milligram of Anti-IL-10R antibody was administered at the time of PZQ treatment (week 6) followed by one more injection on week 7 and week 8. Anti-IL-10RAb (Clone 1B1.3a, BioXCell, New Hampshire, USA) reacts with CD210 (IL-10Rα) the IL-10-specific chain of the IL-10R complex.

### SWAP-specific recall responses *in vitro*

All *in vitro* cultures were performed in complete RPMI 1640 medium supplemented with penicillin (100 U/ml) Streptomycin (100 µg/ml) (GIBCO) and glutamine (4 mM) (GIBCO), plus 10% fetal calf serum (FCS) (GIBCO), unless otherwise stated. Single cell suspensions from lymph nodes or spleens were incubated in quadruplicate at 1×10^6^ cells/well of a round-bottomed 96 well plate in a total of 200 µl at 37°C. Proliferation in response to media (unstimulated control) SWAP antigen (10 µg/ml) or Concanavalin A (1 µg/ml)(Mitogen, positive control) was measured by the addition of 1 µCi of [^3^H] thymidine for the last 18 hours of a 72 hour incubation. Supernatants were collected, after 54 hours of culture, and stored at -80°C for cytokine analysis. [^3^H]-thymidine incorporation was used as an indication of cellular proliferation, with [^3^H] Thymidine incorporation into cellular DNA, captured on glass fiber filter mats (PerkinElmer/Wallac-1450-421) coated with scintillator sheets (PerkinElmer/Wallac-1450-411) and measured in a scintillation counter.

### Polymorphonucelar cell (PMN) and Eosinophil Analysis

EDTA-treated blood was processed for automated counting using Vista Analyzer (Siemens).

#### ELISA

Cytokines were measured by ELISA using Immulon 2HB plates (Thermo) and manufacturers guidelines. Paired capture and detection antibodies from R&D for IL-17A, and IFNγ were used. Capture and detection antibodies for IL-4, IL-5 and IL-10 were purchased from BD Pharmingen. Serum IL-5 and IFNγ was measured using an *in vivo* cytokine capture assay (IVCCA) as previously described [24]. ELISA's plates were washed with 0.05% Tween 20 in PBS (PBST) and blocked with 5% milk in PBST. Recombinant cytokine standards (R&D) were used to assess quantity of cytokines in supernatants using a standard curve, with OD acquired at 405 nm in an ELISA reader.

#### SWAP-specific IgE, IgG1 and IgG2a antibodies were determined by ELISA

Serum samples were diluted in TBS/Tween and wells washed between each incubation step with TBS/Tween. Flat-bottomed 96 well plates were coated with 10 µg/ml of SWAP diluted in carbonate buffer (0.1 mM NaHCO_3_, pH 8.2). After washing, non-specific binding was blocked by incubating wells with 5% BSA (Fraction V, Gibco) in carbonate buffer, for 2 hours at 37°C. Doubling dilutions of serum from 1∶10 to 1∶1280, or as indicated, was then added and plates were incubated overnight at 4°C. For measurement of SWAP-specific-IgE, IgG was first depleted as SWAP-specific-IgG antibodies, which may be in more than 100-fold excess to IgE antibodies, interfere with the detection of SWAP-specific IgE. A 1∶4 dilution of serum in PBS was incubated overnight on a rotator at 4°C with protein-G bound sepharose beads (Pharmacia Biotech) (85 µl, packed beads). IgG-depleted serum was recovered by centrifugation, diluted in TBS/Tween and added to SWAP-coated plates. Plates were incubated with Biotinylated anti-mouse IgE (Clone R35 118, Pharmingen) for 2 hours before Extravidin–alkaline phosphate (Sigma-Aldrich) was added to the plate at 4 µg/ml for 1 hour at 37°C. Finally, *p*-nitrophenyl substrate was added and the reaction was allowed to develop before monitoring at 405 nm. For IgG1, HRP conjugated anti-mouse IgG1 (BD PharMingen) was added at 1∶2000 dilution, HRP conjugated anti-m IgG2a (BD PharMingen) was used at 1∶4000 dilution. Following incubation, 50 µl/well of ABTS peroxidase substrate system (50-62-00, KPL) was added, left to develop and monitored at 405 nm. Total IgE was measured with anti-mouse IgE capture antibody (BD, clone R35-72) and biotinylated anti-mouse IgE detection antibody (BD, clone R35-118,), using a monoclonal IgE standard curve (BD, clone 27-74).

### Flow cytometry

Cells were stained with antibodies diluted in PBS with 0.5% BSA (Sigma-Aldrich) and 0.05% sodium azide (Sigma-Aldrich) for 20 minutes at 4°C. Surface molecule staining (CD3 (BD), CD4 (BD), CD44 (BD), CD25 (eBioscience), B220 (BD), GITR (BioLegend) was carried out on freshly isolated cells. The expression of surface molecules and intracellular molecules (IL-10^gfp^ or foxp3^gfp^) were analyzed on a BD LSR II flow cytometer using FlowJo v.8 software (Tree Star).

### Statistical analysis

Data sets were compared by Mann Whitney test using Prism software v5. Differences were considered significant (*) at P < 0.05.

### Ethics statement

This study was carried out in strict accordance with the recommendations in the Guide for the Care and Use of Laboratory Animals of the National Institutes of Health. The protocol was approved by the Animal Care and Use Committee (ACUC) of the NIAID/NIH (protocol LPD 16E).

## Results

### Praziquantel (PZQ) treatment of *S. mansoni* infected mice increases anti-worm immune responses

**Figure 1:**
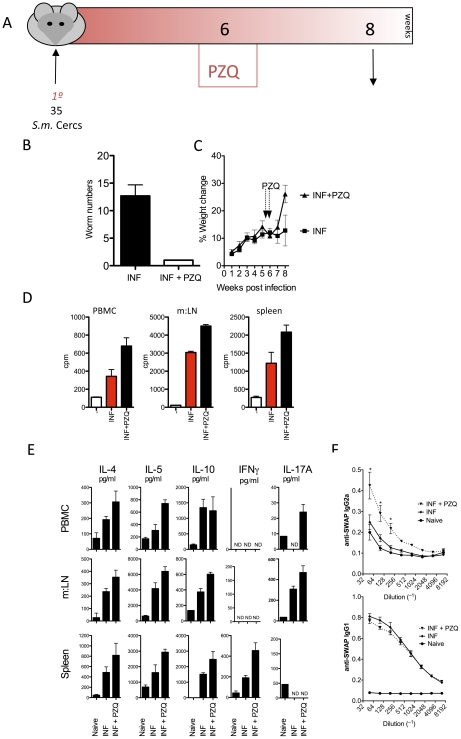
Elevated anti-worm responses post PZQ treatment. C57BL/6 mice were infected with 35 cercariae before treatment with 2 doses of PZQ at week 6. Mice were killed at week 8 for analysis. 10 mice per group were used. Data shown are from one of 3 experiments, with mean ± SEM. (A) Experimental model diagram. (B) Total worm numbers at week 8. 2-weeks post PZQ. (C) Weight change throughout 8-week period. (D) Tritiated [H]^3^ thymidine incorporation in peripheral blood-derived leukocytes, mesenteric lymph nodes and splenocytes following adult worm antigen re-stimulation. (E) Cytokine secretions, measured by ELISA, from adult worm antigen re-stimulation, as in D. (F) Anti-worm antibody responses, measured by ELISA.

Praziquantel (PZQ) continues to be one of the most effective, least expensive and most readily available schistosomicides[5], [6]. Effective removal of adult worms requires synergism between PZQ-associated worm tegument damage [25], [26] and immune-mediated killing [27], [28]. To determine the nature of the immune response responsible for immune mediated killing, we measured anti-worm responses 2 weeks post-PZQ treatment (**Figure 1A**). PZQ-treatment was effective at eliminating adult worms (**Figure 1B**) and led to a spike in weight-gain post treatment (**Figure 1C**). Increased anti-worm specific proliferation was observed in circulating PBMC's, local draining mesenteric lymph nodes (m:LN) and systemically in the spleen following PZQ-treatment (**Figure 1D**). Anti-worm Th2 (IL-4 and IL-5) responses were evident in SWAP-stimulated circulating leukocytes, m:LN and splenocytes (**Figure 1E**) in addition to immunoregulatory IL-10. Similar to observations made here in murine schistosomiasis, schistosome-infected humans treated with PZQ experience a gain in weight [29], an increase in worm-specific cellular proliferation [30], [31] and increase in anti-worm Th2 responses [31]. Anti-worm Th1 (IFNγ) was only detected in the spleen, while evidence of a Th17 response (IL-17A) was only detected in the local m:LN and in the PBMC's of PZQ-treated mice (**Figure 1E**). Thus, following PZQ-treatment, anti-worm Th2, Th1 and Th17 responses were all increased, with statistically significant increases of IL-5, IFNγ, IL-17A and IL-10 in various immunological compartments (**Figure 1E**). Anti-worm IgG2a was also increased following PZQ treatment (**Figure 1F**) with anti-worm IgG1 remaining unchanged and anti-worm IgE undetectable (data not shown). Collectively these data indicate that a mixed anti-worm cytokine, cellular and humoral immune responses were enhanced following PZQ treatment.

### Increased anti-worm responses post-PZQ fails to provide resistance to re-infection

**Figure 2:**
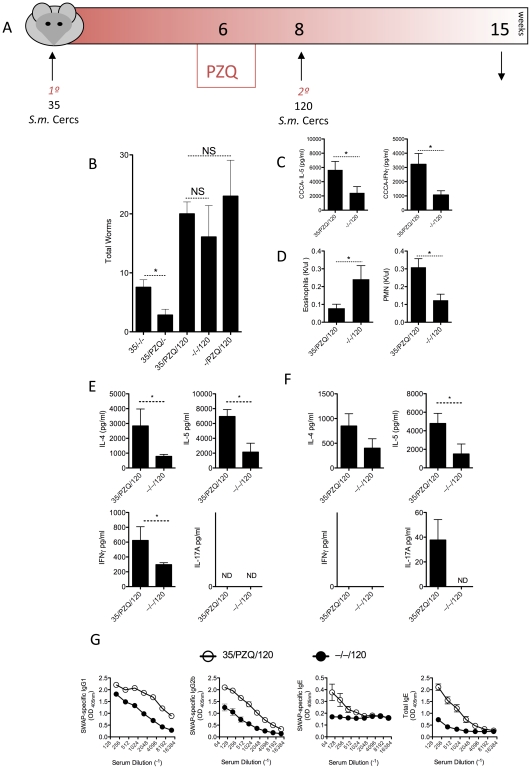
Comparable susceptibility to re-infection despite elevated anti-worm responses in PZQ-treated mice. C57BL/6 mice were infected with 35 cercariae (35/–/–) before treatment with 2 doses of PZQ at week 6 (35/PZQ/–). Mice were re-challenged with 120 cercariae at week 8 (35/PZQ/120), 2-weeks post PZQ treatment and killed at week 15 for analysis. 7–10 mice per group were used. Data shown are from one of 3 experiments, with mean ± SEM. (A) Experimental model diagram. (B)Total worm numbers at week 15. (C) Serum cytokines, IL-5 and IFNγ. (D) Circulating eosinophils and poly-morphonuclear cells. (E) Cytokine secretions, measured by ELISA, from adult worm antigen re-stimulated splenocytes. (F) Cytokine secretions, measured by ELISA, from adult worm antigen re-stimulated mesenteric lymph node cells. (G) Anti-worm antibody responses, measured by ELISA.

Two-weeks post PZQ treatment, when anti-worm immune responses were significantly elevated, compared to infected, untreated mice (**Figure 1**), we re-challenged mice with 120 cercariae to determine if the enhanced anti-worm response would afford any protection against re-infection (**Figure 2A**). Compared to challenged only (–/–/120) and PZQ-treated challenged only control groups (–/PZQ/120), infected and PZQ-treated mice (35/PZQ/120) did not display any protection from a challenge infection (**Figure 2B**)[32]–[33], with similar total worm burden at week 15 (7-weeks post challenge). PZQ-treated mice (35/PZQ/120) mounted a greater systemic IL-5 and IFNγ response (**Figure 2C**) accompanied by elevated circulating polymorphonuclear (PMN) cells but reduced eosinophils (**Figure 2D**), compared to challenge only mice (–/–/120). To determine whether specific anti-worm responses were intact, spleen and mesenteric lymph node cells were re-stimulated with soluble worm antigen (SWAP). Worm-specific IL-4, IL-5 and IFNγ were significantly elevated in the spleen of PZQ-treated mice compared to challenge-only mice (–/–/120, **Figure 2E**) with a similar trend observed in the m:LN's (**Figure 2F**). IL-17A responses, however, were undetectable in the spleen and present at low levels in the m:LN (**Figure 1E, 1F**). Adult worm–specific (IgG1, IgG2b, IgE) and total IgE antibody titers were significantly elevated in PZQ-treated mice compared to challenge-only mice (**Figure 2G**). However, despite the development of significantly increased systemic cytokine responses, mobilization of granulocytes and the maturation of worm-specific cytokine and antibody responses, there was no resistance to re-infection.

### Increased IL-10^+^CD4^+^CD44^+^CD25^+^GITR^+^ cells post-PZQ

**Figure 3:**
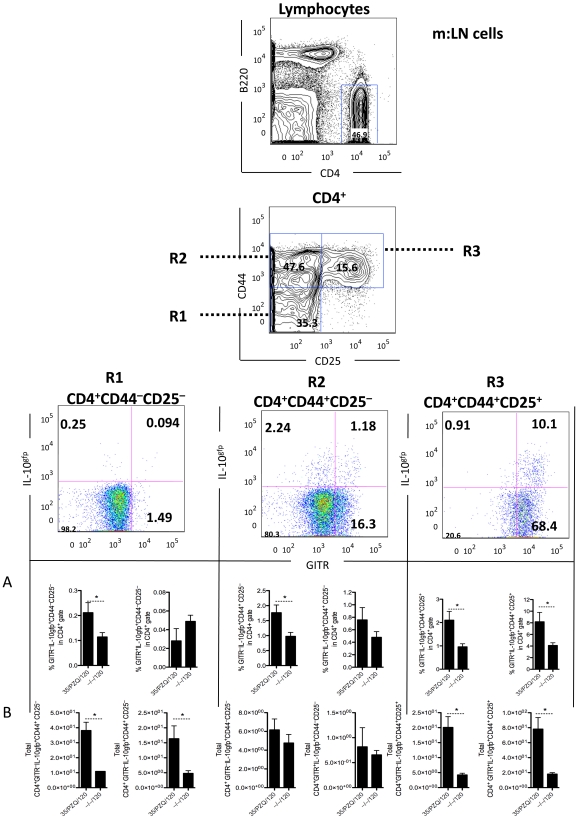
Elevated CD4^+^CD44^+^CD25^+^GITR^+^IL-10gfp^+^ cells in PZQ-treated mice. C57BL/6 mice were infected as in Figure 2, with cells isolated from the mesenteric lymph nodes at necropsy. Cells were stained, as in methods and analyzed with FlowJo software. Data shown are mean ± SEM from one of 2 experiments, with 5 mice per group. (A) % of cells in CD4 gate. (B) Total number of cells in mesenteric lymph nodes (x10^3^).

**Figure 4:**
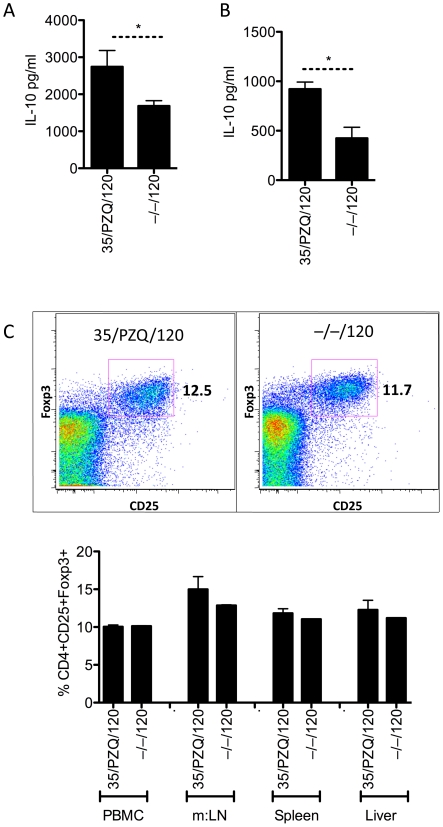
Elevated worm-specific IL-10 from spleen and mesenteric lymphnodes, with unchanged Foxp3+ cells. C57BL/6 mice were infected as in Figure 2, with cells recovered at necropsy. Data shown are mean ± SEM from one of 2 experiments, with 5 mice per group. (A) IL-10 secretions, measured by ELISA, from adult worm antigen re-stimulated splenocytes. (B) IL-10 secretions, measured by ELISA, from adult worm antigen re-stimulated mesenteric lymph node cells. (C) Foxp3 and CD25 stained CD4+ lymphocytes from peripheral blood, mesenteric lymph nodes (m:LN), spleen and liver.

In addition to the effector arm of the immune response (**Figure 2**), we analyzed the development of immunoregulatory responses following PZQ-treatment and re-challenge. Using IL-10^gfp^ reporter mice in conjunction with several immunoregulatory co-receptors, we profiled the regulatory T cell responses. At the time of re-challenge (week 8, data not shown) and at week 15, we observed an expansion of IL-10-producing cells (**Figure S1**and **Figure 3**). Using multi-color flow cytometry, we identified a major population of IL-10-producing cells within the CD4^+^ lymphocyte population. We further delineated a sub-population of CD4^+^CD44^+^CD25^+^GITR^+^ cells (**R3**, **Figure 3**), which were increased proportionally (**Figure 3A**) and in total number (**Figure 3B**) in the mesenteric lymph nodes, in addition to other IL-10-producing populations within the CD4^+^CD44^+^CD25^–^GITR^+/–^ gates (**R2**, **Figure 3**). Similar IL-10-producing populations were observed in the liver, peripheral blood and spleen (**Figure S2 A, B, C**). Of particular interest, we observed a significant increase in IL-10-prodcuing cells in PZQ-treated mice, compared to challenge-only mice (**Figure 3**). Together with data presented in **figure 2**, the increased effector responses (**Figure 2**) appeared to be mirrored by a significant expansion of IL-10-producing cells (**Figure 3**). Furthermore, we also observed a significant increase in worm-specific IL-10 production in the spleen (**Figure 4A**) and m:LN (**Figure 4B**) of PZQ-treated mice. The frequency of CD4^+^CD25^+^Foxp3^+^ cells however did not change significantly in any of the sites examined (**Figure 4C**), neither were their differences in other inhibitory receptors BTLA, PD1, CTLA-4, or LAG3 (data not shown). Thus, in addition to the development of significant anti-worm Th1, Th2 and Th17-associated effector responses in PZQ-treated mice (**Figure 2**), a noteworthy increase in CD4^+^CD44^+^CD25^+^GITR^+^IL-10gfp^+^ T cells were also observed in similar immunological sites.

### IL-10R blockade during PZQ treatment increases resistance to re-infection

**Figure 5:**
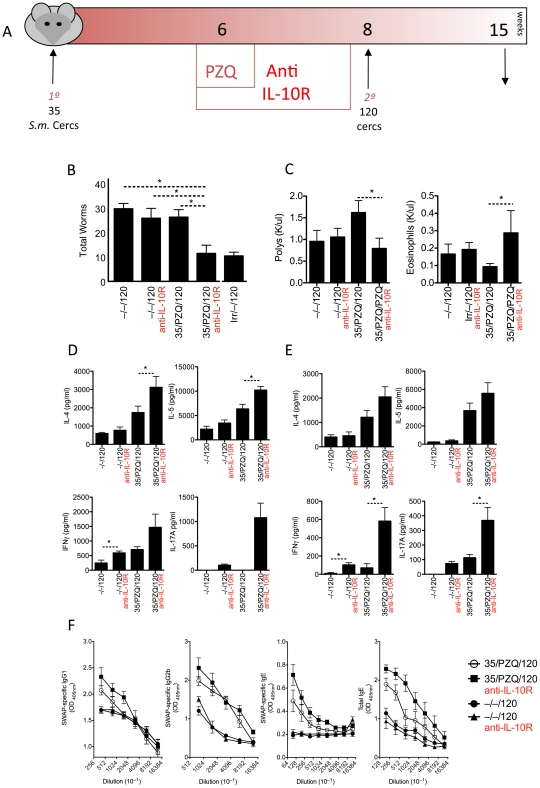
IL-10-blockade establishes resistance to re-infection. C57BL/6 mice were infected as in Figure 2, with a sub group of mice treated with anti-IL-10RAb (1 mg/mouse at the start of PZQ treatment- wk6, wk7 and wk 8). Data shown are mean ± SEM from one of 3 experiments, with 7 mice per group. (A) Experimental model diagram. (B) Total worm numbers at week 15. (C) Circulating eosinophils and poly-morphonuclear cells. (D) Cytokine secretions, measured by ELISA, from adult worm antigen re-stimulated splenocytes. (E) Cytokine secretions, measured by ELISA, from adult worm antigen re-stimulated mesenteric lymph node cells. (F) Anti-worm antibody responses, measured by ELISA.

We hypothesized that the lack of resistance to re-infection, in the face of significant anti-worm immune responses, was due to increased IL-10, which could be inhibiting protective anti-worm immunity. We tested this hypothesis by blocking IL-10 signaling with anti-IL-10 receptor blockade from week 6 (at the time of PZQ treatment) to week 8 (time of challenge infection) (**Figure 5A**). Significantly fewer worms were recovered at week 15 in mice treated with anti-IL-10R Ab (35/PZQ/120 + anti-IL-10R), compared to challenge-only (–/–/120), challenge-only and anti-IL-10R Ab treatment (–/–/120 + anti-IL-10R) or PZQ-treated mice with control antibody (35/PZQ/120)(**Figure 5B**). Moreover, IL-10R blockade during PZQ treatment afforded similar protection as mice given an irradiated cercariae vaccination (Irr/–/120), the gold standard for vaccine-induced immunity. These data indicate that IL-10 impedes the development, or effector mechanisms, of resistance to re-infection after treatment. Correlating with increased resistance was a reduction in circulating neutrophils and an increase in eosinophils (**Figure 5C**). Anti-worm Th2 (IL-4 and IL-5), as well as Th1 (IFNγ) and Th17 (IL-17A) responses in the spleen (**Figure 5D**) and m:LN (**Figure 5E**) were also elevated in resistant mice. Correlating with the broadly enhanced T helper responses in anti-IL-10R Ab treated mice, anti-worm IgG1, IgG2b and IgE isotypes were also increased (**Figure 5F**). Anti-schistosomula responses in the spleen and m:LN were increased following PZQ treatment, however there was no significant change with the addition of anti-IL-10R blockade (**Figure S3**).

**Figure 6:**
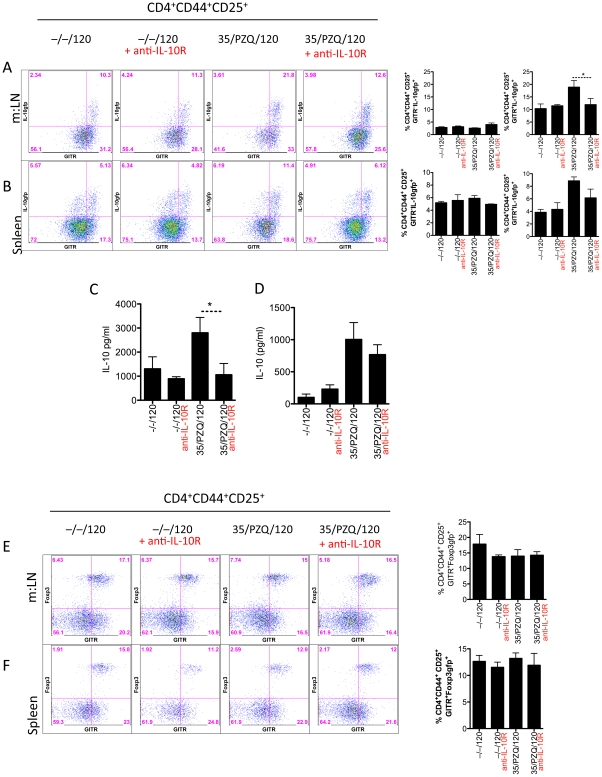
IL-10 blockade, reduces IL-10 producing cells, IL-10 secretion from spleen and mesenteric lymph nodes, with unchanged Foxp3+ cells. C57BL/6 or Foxp3^gfp^-reporter mice were infected and treated, as in Figure 5, with cells recovered at necropsy. Data shown are mean ± SEM from one of 2 experiments, with 5 mice per group. (A) GITR^+^IL-10gfp^+^ mesenteric lymph nodes cells within the CD4^+^CD44^+^CD25^+^ population. (B) GITR^+^IL-10gfp^+^ splenocytes within the CD4^+^CD44^+^CD25^+^ population. (C) IL-10 secretions, measured by ELISA, from adult worm antigen re-stimulated. mesenteric lymph node cells. (D) IL-10 secretions, measured by ELISA, from adult worm antigen re-stimulated splenocytes. (E) GITR^+^Foxp3^+^ mesenteric lymph nodes cells within the CD4^+^CD44^+^CD25^+^ population, using Foxp3^gfp^ reporter mice. (F) GITR^+^Foxp3^+^ splenocytes within the CD4^+^CD44^+^CD25^+^ population, using Foxp3^gfp^ reporter mice.

Animals treated with anti-IL-10R antibodies had a reduced frequency of IL-10-producing CD4^+^CD44^+^CD25^+^GITR^+^ cells in the m:LN (**Figure 6A**) and, to a lesser extent, in the spleen (**Figure 6B**), suggesting that IL-10 signaling may promote the development of IL-10-producing CD4+ T cells. *In vitro* re-stimulation with soluble worm antigen also highlighted the reduced IL-10 responses in anti-IL-10R Ab treated mice (**Figure 6C, D**). Irrespective of IL-10R blockade or PZQ treatment, the frequency of CD4^+^CD44^+^CD25^+^GITR^+^Foxp3^+^ cells in the spleen or m:LN did not change. Approximately 50% of GITR^+^ cells in the m:LN were also Foxp3^+^ and at this time we cannot completely exclude the role of Foxp3. However, given the relative unaffected frequencies of Foxp3^+^ cells in all experimental conditions, we consider IL-10 to be the most important inhibitor of resistance to re-infection. Taken together, these data indicate that blocking IL-10 signaling during PZQ treatment can accelerate resistance to re-infection. A wide range of immunological parameters were elevated in resistant mice, including anti-worm Th1, Th2 and Th17 responses as well as a mixed antibody response. Although a single immunological parameter did not stand out and correlate with resistance, a mixed response may indeed be the correlate of protection in this system.

## Discussion

In this study we have demonstrated that following PZQ treatment, IL-10 inhibits the development of protective immunity to secondary schistosome infection. We observed an elevated anti-worm immune responses following PZQ treatment alone, however this failed to confer any protection to a secondary challenge infection, as previously observed [33]. This was largely due to the inhibitory effects of IL-10, as blockade of IL-10 combined with PZQ treatment raised protective immunity from 0% to more than 50% when compared with PZQ treatment alone.

Although still debated, resistance to re-infection in humans appears to develop following repeated cycles of treatment and re-exposure. Exposure history can only be estimated, and therefore the notion that many rounds of re-infection are required before resistance develops has stood for many years. This has led to an age-dependent resistance model [17], [34], with suggestions that in addition to several rounds of exposure, puberty and hormones may also influence resistance to re-infection [35], [36]. Recent evidence however, suggests that resistance is not strictly tied to age, but rather resistance is a product of exposure and curative treatment cycles, combined with changes in the immunological status of the host [18], [37]. In a recent study, patients rapidly, gradually, or never developed resistance to re-infection throughout a 5-year prospective treatment- re-exposure study. These findings suggested to us that resistance might be primarily immunologically determined, rather than being strictly dependent on age and exposure history.

Following PZQ treatment, *S. mansoni*-infected patients have been shown to exhibit elevated IL-4, IL-5 and IL-13 [19], [20] responses and increased numbers of circulating eosinophils [38]. Colley and colleagues [30] have also observed elevated anti-worm responses in schistosome infected Egyptians up to 2-years following PZQ treatment. We observed a similar increase in parasite-specific T cell proliferation, cytokine secretion, and antibody production in infected mice following PZQ treatment. However, despite exhibiting significantly elevated anti-worm responses, the mice remained fully susceptible to a secondary challenge infection. Mitchell and colleagues also investigated this phenomenon and found that immunizing mice with adult worm antigens in combination with PZQ treatment was still incapable of conferring protection from re-infection [39]. A large proportion of drug treated patients also remain highly susceptible to reinfection [30]. The mechanism behind the failure to generate a protective recall response following a cleared primary infection remains unclear. The current hypothesis from human studies suggests that several treatment and re-exposure cycles are needed, which triggers the release of worm antigens[40] that repeatedly prime and boost the immune response, analogous to the immunity achieved following multiple vaccinations. Several rounds of treatment and re-infection likely mimic and accelerate the acquisition of acquired immunity, which would otherwise take years to develop [15]. So, the question remains - why do infected and treated hosts develop increased anti-worm immune responses but remain susceptible to re-infection?

We designed experiments to avoid the influence of egg-induced liver inflammation when non-specific resistance can develop[41]. The cost of such experimental design however, does pose a limitation on translating our findings to human infections, where diagnosis is often based upon egg detection. We hypothesized that opposing immunoregulatory responses were developing in parallel with the effector response and inhibiting the activation of effective anti-worm immunity. In support of this hypothesis, we observed a marked increase in IL-10 production and identified a population of putative regulatory T cells (CD4+CD44+CD25+GITR+IL-10gfp+) following PZQ treatment. Although we did not observe any change in Foxp3+ cells following PZQ treatment, we cannot rule out the possibility that the IL-10-producing T cells are a subset of Foxp3+ natural regulatory T cells or recently activated effector T cells. An increased IL-10 response has also been observed in PZQ-treated humans, which similarly paralleled an elevated anti-worm Th2 response [20]. Longitudinal immunological studies conducted in schistosome endemic regions have also strongly implicated a negative regulatory role for IL-10 in the development of resistance to re-infection in humans [42], [43]. Despite developing enhanced parasite-specific IL-5 responses following treatment, Van den Bigelaar and colleagues identified a concurrent increase in parasite-specific IL-10, and proposed that IL-10 was a major risk factor for re-infection. Similarly, Leenstra and colleagues [43] found that elevated IL-10 predicted a decrease in time to re-infection with several reports describing inverse correlations between IL-10 and *S. mansoni* [44] or *S. haematobium* [45] infection intensity. These data support the concept that IL-10 functions as an important regulator of protective immunity and resistance to re-infection, which is consistent with its well established role as a suppressor of immunopathology during infection with *S. mansoni* [46], [47], [48], [49], [50], [51], [52], [53], [54], [55], [56], [57]. IL-10 has also been shown to suppress *S. manoni* egg and worm-specific human PBMC proliferation and cytokine production *in vitro* [58], [59], [60], [61]. However, whether IL-10 obstructs the development of resistance to re-infection remained unclear.

We hypothesized that IL-10 was suppressing both anti-worm and anti-larval responses and was responsible for the failure to develop resistance [62], [63]. We tested the impact of IL-10 in the development of resistance in a therapeutic model, combining anti-IL-10R Ab treatment with PZQ treatment. IL-10-blockade during PZQ treatment further increased anti-worm immune responses, and afforded significant protection from re-infection. Anti-larval responses were not directly measured in this study and although there may be cross-reactivity between adult and larval epitopes, increased resistance correlated with increased anti-adult immune parameters. The level of protection following combined PZQ treatment and IL-10 blockade equaled that achieved by the irradiated cercariae vaccination, which serves as the ‘gold standard’ for vaccine induced immunity. This observation is supported from other studies suggesting that immunoregulatory responses, in particular IL-10, can actively impede the development of immunity to infection either naturally [64], [65], [66], [67], or following vaccination [68], [69], [70], [71], [72], [73]. Whether the Glucocorticoid-induced TNFR-related protein (GITR), which is expressed on these cells, functions as a proliferative [74], cytokine enhancing [75], or inhibitory receptor is currently unknown. Nevertheless, it is clear from our studies that IL-10 expressed in GITR^+^CD4^+^ T cells and potentially other cell types[53], restricts the type and magnitude of the protective immune response following treatment with PZQ. Thus, combining IL-10R blockade with chemotherapy may accelerate the development of protective immunity in otherwise permissive hosts.

It has previously been reported that resistance to re-infection in mice can be achieved through physiological means, as mentioned above. Wilson and colleagues [76] demonstrated that the development of anastomoses precludes parasite maturation and migration, with reduced adult worm establishment. Although we cannot rule this out in our system, we believe there to be an immunological mechanism of resistance following IL-10R blockade. Many immunological correlates of resistance to re-infection in humans have been reported including IL-5 [17], [77] and eosinophilia [78], [79], mast cells [80] and IFNγ [77]. Antibody responses, in particular anti-IgE adult worm antibodies [17], [78], [81], [82], [83], [84], [85], [86] and CD23^hi^ B cell responses [87] have also been described. In our studies, blockade of IL-10 in conjunction with PZQ treatment led to an increase in eosinophilia and adult worm-specific IL-4, IL-5, IFNγ and IL-17A. Anti-worm-specific IgG1, IgG2b and IgE were also elevated, providing evidence of an exaggerated but mixed cytokine profile, which was shown previously to improve vaccine-induced immunity to *S. mansoni* by boosting both humoral and cell-mediated immune responses against the parasite[62]. Whether larval stages (cercariae, early and late stage schistosomula) are the targets of protective immunity with anti-IL-10R mAb + PZQ treatment are currently unknown. Greater protection from schistosome infection has been observed in IL-10-deficient mice given an irradiated cercariae vaccine [62]. In this scenario a mixed Th1/Th2 response was observed. Furthermore, whether a single immunodominant antigen is responsible for the protection is also not known. Conceivably, IL-10 blockade may allow a wider repertoire of antigens with greater magnitude and diversity. The impact of IL-10 on antigen repertoire and different classes of immune responses with respect to resistance to re-infection should be the subject of future studies if this regimen is to move successfully into patients.

In conclusion, this study demonstrates that IL-10, derived predominantly from CD4+ lymphocytes, hampers the development of critical effector mechanisms that mediate resistance to schistosome infection following treatment. These observations suggest that immunomodulators delivered in combination with PZQ treatment may be required to generate the robust and mixed humoral and cell-mediated immune response that is required to prevent reinfection with schistosomes.

## Supporting Information

Figure S1 **Elevated CD4^+^IL-10gfp^+^ cells, with minor increases in B220^+^IL-10gfp^+^ and CD8^+^IL-10gfp^+^ cells in PZQ-treated mice.** C57BL/6 mice were infected as in Figure 2, with cells isolated from the spleen, stained as in methods and analyzed with FlowJo software. Data shown are mean ± SEM from one of 2 experiments, with 5 mice per group.
(TIFF)

Figure S2 **Elevated CD4^+^CD44^+^CD25^+^GITR^+^IL-10gfp^+^ cells in PZQ-treated mice.** C57BL/6 mice were infected as in Figure 2 with cells isolated, stained as in methods and analyzed with FlowJo software. Data shown are representative from one of 2 experiments, with 5 mice per group. (A) Liver. (B) Peripheral blood.(C) Spleen.
(TIFF)

Figure S3 **Anti-Schistosomula cytokine responses within the spleen and mesenteric lymph nodes (m:LN).** C57BL/6 mice were infected as in Figure 5, with cells isolated from the spleen and m:LN at necropsy. Cytokine secretions were measured by ELISA, from schistosomula antigen re-stimulated cells. Data shown are mean ± SEM from one of 2 experiments, with 5 mice per group
(TIFF)
