# Supplementary figures and images for: IL-10 Blocks the Development of Resistance to Re-Infection with Schistosoma mansoni
Source: PLoS Pathog. 2011 Aug 4;7(8):e1002171. doi: 10.1371/journal.ppat.1002171 (PMC3150278; doi:10.1371/journal.ppat.1002171)

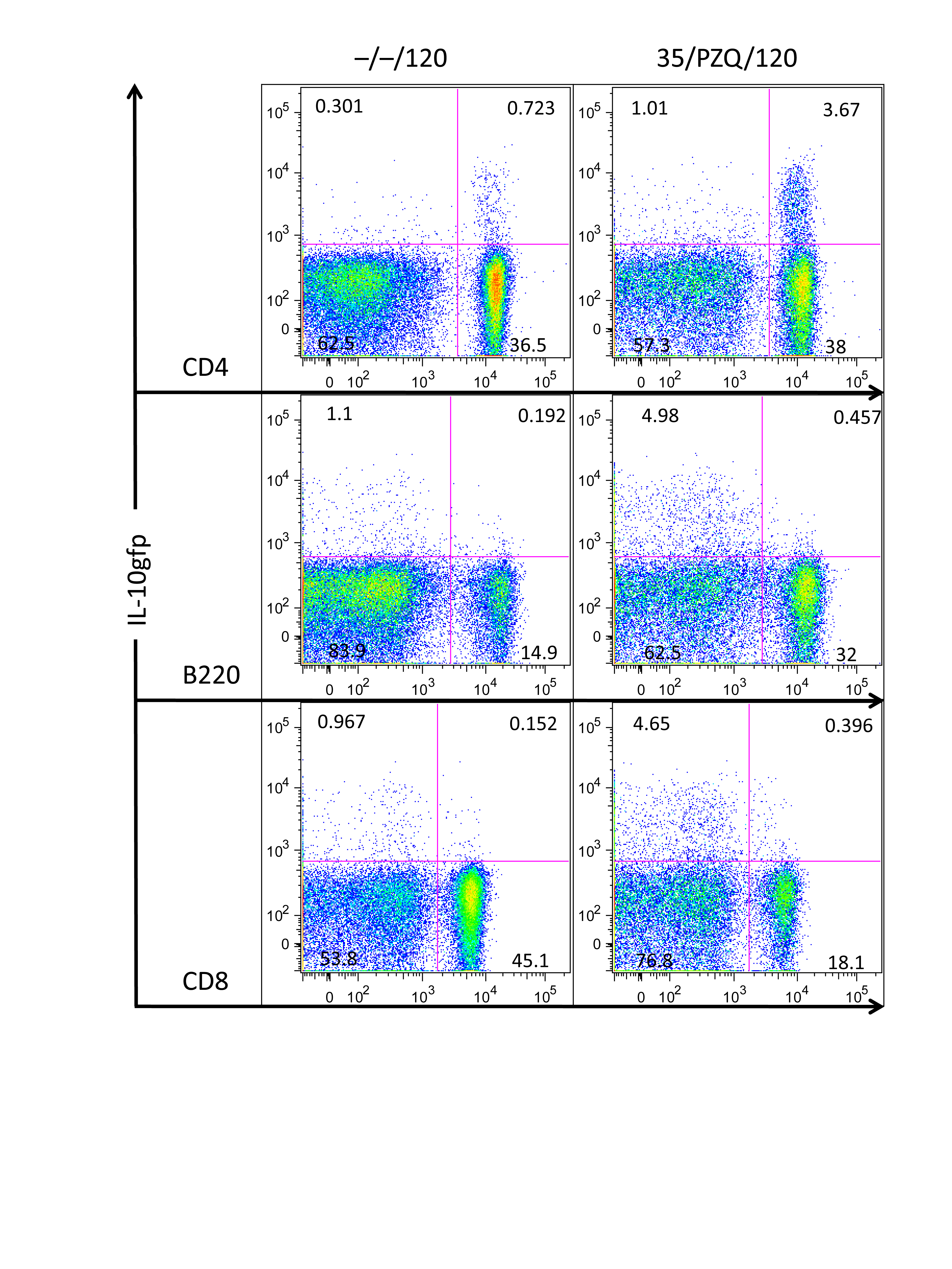

Supplement: Figure S1 — Elevated CD4+IL-10gfp+ cells, with minor increases in B220+IL-10gfp+ and CD8+IL-10gfp+ cells in PZQ-treated mice. C57BL/6 mice were infected as in Figure 2, with cells isolated from the spleen, stained as in methods and analyzed with FlowJo software. Data shown are mean ± SEM from one of 2 experiments, with 5 mice per group. (TIFF) [file ppat.1002171.s001.tiff]

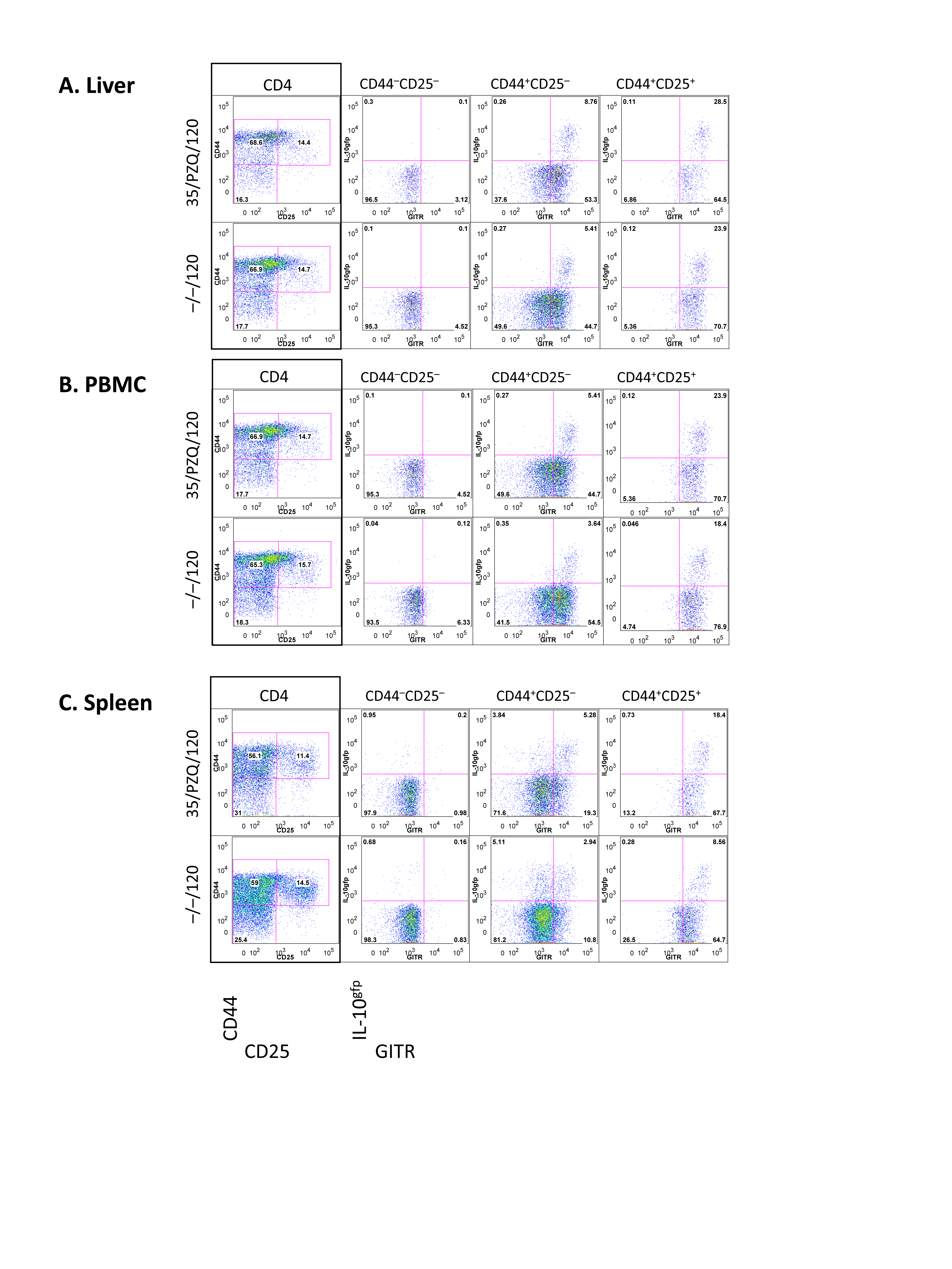

Supplement: Figure S2 — Elevated CD4+CD44+CD25+GITR+IL-10gfp+ cells in PZQ-treated mice. C57BL/6 mice were infected as in Figure 2 with cells isolated, stained as in methods and analyzed with FlowJo software. Data shown are representative from one of 2 experiments, with 5 mice per group. (A) Liver. (B) Peripheral blood.(C) Spleen. (TIFF) [file ppat.1002171.s002.tiff]

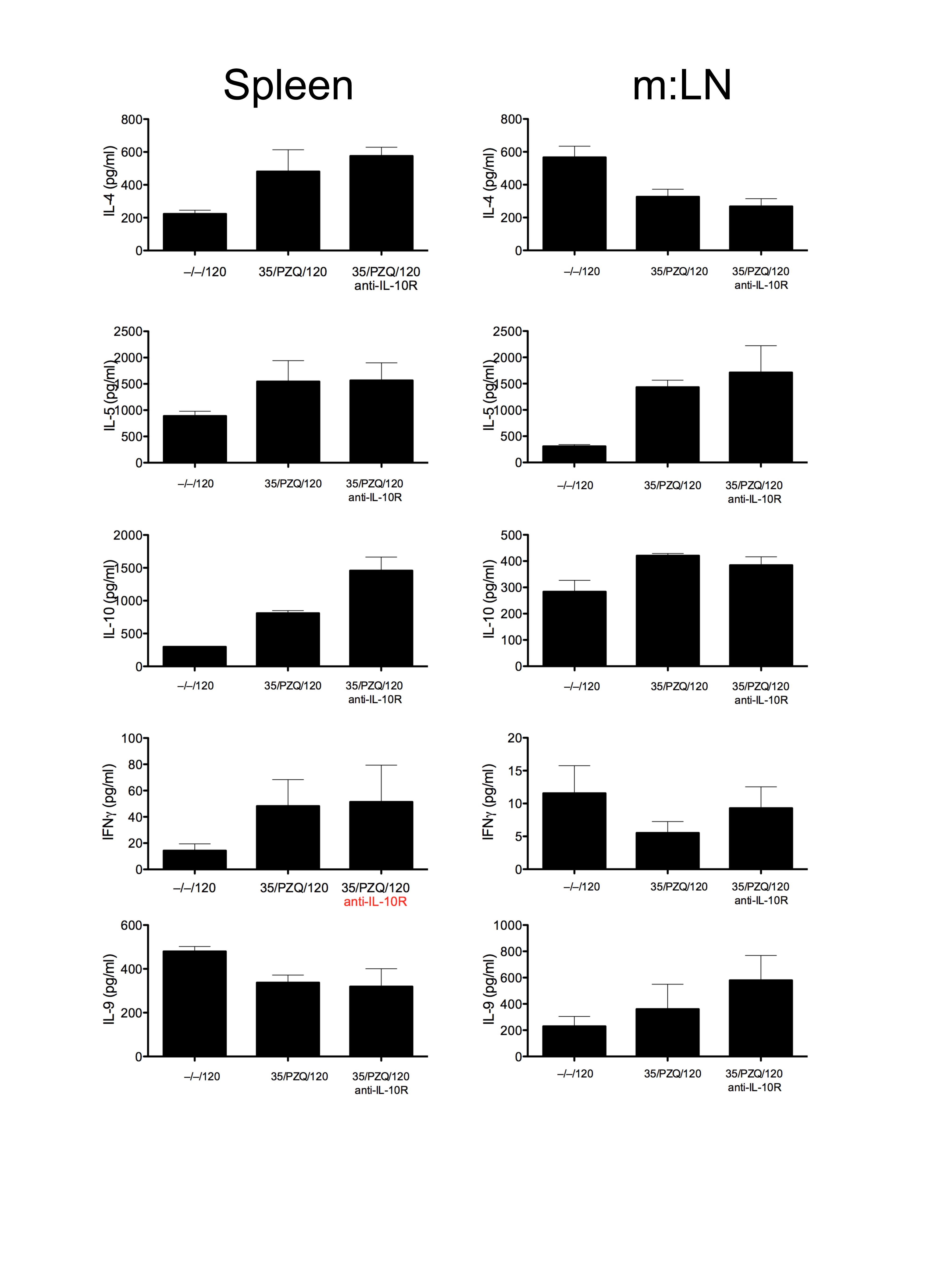

Supplement: Figure S3 — Anti-Schistosomula cytokine responses within the spleen and mesenteric lymph nodes (m:LN). C57BL/6 mice were infected as in Figure 5, with cells isolated from the spleen and m:LN at necropsy. Cytokine secretions were measured by ELISA, from schistosomula antigen re-stimulated cells. Data shown are mean ± SEM from one of 2 experiments, with 5 mice per group (TIFF) [file ppat.1002171.s003.tiff]
